# Genome Sequences of *Acholeplasma laidlawii* Strains with Increased Resistance to Tetracycline and Melittin

**DOI:** 10.1128/genomeA.01446-17

**Published:** 2018-01-11

**Authors:** Natalia B. Baranova, Tatyana Y. Malygina, Elena S. Medvedeva, Eugenia A. Boulygina, Maria N. Siniagina, Mohamed Amine Dramchini, Reshad Akbor Prottoy, Alexey A. Mouzykantov, Marina N. Davydova, Olga A. Chernova, Vladislav M. Chernov

**Affiliations:** a Kazan Institute of Biochemistry and Biophysics, Kazan Scientific Centre of the Russian Academy of Sciences, Kazan, Russia; b Institute of Fundamental Medicine and Biology, Kazan (Volga Region) Federal University, Kazan, Russia

## Abstract

*Acholeplasma laidlawii* is a well-suited model for studying the molecular basis for adapting mollicutes to environmental conditions. Here, we present the whole-genome sequences of two strains of *A. laidlawii* with increased resistance to tetracycline and melittin.

## GENOME ANNOUNCEMENT

The recommended approach to suppress and eliminate bacteria belonging to the class *Mollicutes*—the parasites of plants, animals, and humans, as well as the main contaminants of cell cultures (1, 2)—is antibiotic therapy associated with the use of fluoroquinolones, tetracyclines, and macrolides (3, 4). An alternative method is related to antimicrobial peptides, including melittin, a peptide from honey bee venom (5, 6). *Acholeplasma laidlawii*, a ubiquitous mollicute, is a well-suited model for studying the molecular basis for adapting mollicutes to environmental conditions, including the development of antibiotic resistance *in vitro* (7–10). Previously, we presented the whole-genome sequences of *A. laidlawii* strains with different sensitivity to ciprofloxacin (11). The genomes of two strains of *A. laidlawii* with increased resistance to tetracycline (PG8R_Tet_) and melittin (PG8R_Mel_), which are derivatives of the PG8B strain (GenBank accession number LVCP00000000), were sequenced in this study.

DNA from cells of the *A. laidlawii* strains was extracted using the phenol extraction method (12). The DNA concentration was determined using a Qubit version 2.0 fluorometer (Invitrogen). The fragmentation was carried out using a Covaris S220 ultrasonic disintegrator (Thermo Fisher Scientific). After sonication, the samples were cleaned with AMPure magnetic particle beads (Beckman Coulter, Inc.). The libraries were prepared with an NEBNext Ultra II kit (New England Biolabs) according to the manufacturer’s instructions. The quality analysis of the DNA libraries was performed on a 2100 Bioanalyzer instrument (Agilent). The whole-genome sequencing of the obtained libraries was performed on the MiSeq platform (Illumina, USA) using 150-bp paired-end reads. The *de novo* assembly of the received reads was performed using the SPAdes version 3.7.0 genome assembler (13). Alignment to the reference genome of *A. laidlawii* PG-8A (GenBank accession number CP000896) and annotation of single nucleotide polymorphisms (SNPs) were performed using Bowtie2 software (14), SAMtools (15), and SnpEff version 3.3 (16). Gene prediction and annotation were performed using the NCBI Prokaryotic Genome Annotation Pipeline (17).

Mutations in the genes associated with the development of tetracycline resistance in different microorganisms were not found in the genome of *A. laidlawii* PG8R_Tet_. SNPs in genes coding membrane and efflux proteins as well as proteases were found in the genome of *A. laidlawii* PG8R_Mel_. It is assumed that these proteins are associated with the development of resistance to antimicrobial peptides in different microorganisms (18). In addition, SNPs in PG8R_Mel_ as well as in PG8R_Tet_ were found in many genes, and their involvement in antibiotic resistance remains to be elucidated. Some SNPs found in PG8R_Tet_ and PG8R_Mel_ were also detected in *A. laidlawii* strain PG8R_10_, which has increased resistance to ciprofloxacin (GenBank accession number LXYB01000000).

The whole-genome sequences of *A. laidlawii* strains PG8R_Tet_, PG8R_Mel_, and PG8R_10_ with differential sensitivity to tetracycline, melittin, and ciprofloxacin, can be used further to determine the molecular basis for adapting mollicutes to antimicrobial agents.

### Accession number(s).

The whole-genome shotgun projects of PG8R_Tet_ and PG8R_Mel_ have been deposited in DDBJ/ENA/GenBank under the accession numbers NELO00000000 and NELN00000000, respectively. The versions described in this paper are the second versions, NELO02000000 and NELN02000000.
